# PLGA Microspheres with Alginate-Coated Large Pores for the Formulation of an Injectable Depot of Donepezil Hydrochloride

**DOI:** 10.3390/pharmaceutics12040311

**Published:** 2020-04-01

**Authors:** Dohyun Kim, Tae Hee Han, Seong-Chul Hong, Sun Jae Park, Yong Hak Lee, Hyeongmin Kim, Minwoo Park, Jaehwi Lee

**Affiliations:** College of Pharmacy, Chung-Ang University, Seoul 06974, Korea; dylan13@naver.com (D.K.); hanths@hanmail.net (T.H.H.); shotgun30@naver.com (S.-C.H.); sunjae1993@naver.com (S.J.P.); lyh7685@naver.com (Y.H.L.); hm.kim8905@gmail.com (H.K.); qkralsdn9310@naver.com (M.P.)

**Keywords:** donepezil depot, sustained release, porous microsphere, pore size, porosity, drug loading, burst release, pore closing, convolution

## Abstract

As the main symptom of Alzheimer’s disease-related dementia is memory loss, patient compliance for donepezil hydrochloride (donepezil), administered as once-daily oral formulations, is poor. Thus, we aimed to design poly(lactic-*co*-glycolic acid) (PLGA) microspheres (MS) with alginate-coated large pores as an injectable depot of donepezil exhibiting sustained release over 2–3 weeks. The PLGA MS with large pores could provide large space for loading drugs with high loading capacity, and thereby sufficient amounts of drugs were considered to be delivered with minimal use of PLGA MS being injected. However, initial burst release of donepezil from the porous PLGA MS was observed. To reduce this initial burst release, the surface pores were closed with calcium alginate coating using a spray-ionotropic gelation method. The final pore-closed PLGA MS showed in vitro sustained release for approximately 3 weeks, and the initial burst release was remarkably decreased by the calcium alginate coating. In the prediction of plasma drug concentration profiles using convolution method, the mean residence time of the pore-closed PLGA MS was 2.7-fold longer than that of the porous PLGA MS. Therefore, our results reveal that our pore-closed PLGA MS formulation is a promising candidate for the treatment of dementia with high patient compliance.

## 1. Introduction

Alzheimer’s disease (AD) is a chronic neurodegenerative disease that is the cause of 60%–70% of cases of dementia [1]. As a deficiency of the neurotransmitter acetylcholine in the brain is closely associated with the cognitive impairment of the patients with AD [2,3,4,5], acetylcholine esterase inhibitors are commonly used to improve the symptoms of AD. Donepezil hydrochloride (donepezil), a piperidine-based acetylcholine esterase inhibitor, is one of the most frequently used acetylcholine esterase inhibitors because of its low hepatotoxicity, better tolerance, and lower incidence of adverse events compared with other drugs such as tacrine, rivastigmine, and galantamine [6,7].

Commercial products of donepezil are primarily available as conventional oral dosage forms such as tablets and capsules [8,9]. AD patients need to take the tablet or capsule once a day [10]. However, the memory loss caused by the disease leads to poor compliance with the daily dosing regimen of donepezil; in fact, approximately one-third of AD patients were found to be non-compliant with taking their donepezil tablets regularly [11,12]. Such inadequate oral administration of donepezil tablets or capsules may cause large and frequent fluctuations in plasma drug concentrations and consequently increase the incidence of adverse effects of donepezil such as nausea, vomiting, and diarrhea [13]. Therefore, it is necessary to develop an alternative pharmaceutical formulation for donepezil that can reduce the dosing frequency and adverse effects of the drug.

Injectable depot systems could be used to overcome the limitations of the oral dosage forms of donepezil, owing to their advantages such as low dosing frequency, less fluctuation in drug plasma concentration, rapid onset of action, and high bioavailability [14,15]. Diverse injectable depot systems have been investigated, such as injectable suspensions [16], emulsions [17], micro-/nanospheres [18,19,20], and liposomes [21]. Among them, poly(lactic-*co*-glycolic acid) (PLGA) microspheres (MS) with an open porous structure are very promising because they can accommodate drugs efficiently in their pore spaces, leading to high drug encapsulation efficiency (EE) and loading capacity (LC), which is essential for prolonged drug release from injectable depot systems. PLGA has been extensively used to prepare the porous MS because of its excellent biocompatibility and predictable degradation rate that is based on the molecular weight and composition of the polymer [22,23]. In addition, drug release kinetics can be controlled by adjusting the pore size and porosity of the PLGA MS. However, the initial burst drug release from porous MS causes the drug concentration to exceed toxic limits and shorten the duration for which the drug concentration is maintained in the therapeutic window. Thus, the controllability of drug release profiles from porous MS needs to be improved.

Closing the pores of MS could help to avoid the initial burst drug release and control the drug release behavior from the MS. For this purpose, some researchers have used solvents that partially dissolve the MS [24,25]. However, this approach could cause the leakage of drugs from the large pore space into the surrounding media during the pore-closing process, leading to poor drug loading in the MS. Moreover, the use of solvents could easily damage the porous structure of the MS, resulting in drug leakage and low LC. Regarding the use of polymers to coat the PLGA microspheres, alginate and chitosan have been primarily employed mainly due to their good biocompatibility and biodegradability [23,26]. One of the big differences of alginate and chitosan is that alginate is an anionic polymer whereas chitosan is a cationic polymer. The alginate polymer matrices, owing to their negative charges, successfully retard the release of cationic drugs such as donepezil hydrochloride through electrostatic interaction [27,28,29]. Furthermore, they can undergo mild gelation by the addition of divalent cations such as calcium ions [30], and therefore the drug release kinetics from the pores could be precisely controlled by controlling the thickness of the calcium alginate coating. Thus, alginate seems to be adequate for our study as a pore-closing agent.

Therefore, in this study, we aimed to design PLGA MS with relatively big pores on their surface closed by calcium alginate coating as injectable depot systems for prolonged release of donepezil, leading to the development of a novel delivery system and high patient compliance. Multiple studies have been performed to fabricate the PLGA MS with large pores for delivering large substances such as therapeutic cells and peptides because of its large pore space [31,32,33]. In our previous study [34], we also designed PLGA MS for the purpose of loading therapeutic cells, which exhibited large pore sizes with a diameter greater than 15 µm. The PLGA MS with large pores designed for loading therapeutic cells is considered advantageous as it can provide more space to load the drug, thereby leading to a decrease in the amount of PLGA MS to be injected. However, when big pores remain open on the surface of PLGA MS, drug release would not be controlled. Thus, we employed alginate to coat the surface of PLGA MS with large pores to avoid an initial burst release and control drug release. Large pores on the surface of the PLGA MS were expected to be closed with alginate coating by spraying the MS suspended in aqueous alginate solutions into aqueous calcium solutions [35,36]. To demonstrate the sustained release profiles of the PLGA MS formulation, in vitro drug release profiles as a function of the amount of alginate used were evaluated, and plasma drug concentration profiles were predicted by using convolution method. There have been many reports studying the PLGA MS for the sustained release injection [8,37,38]. However, to the best of our knowledge, this is the first study that involved designing the PLGA MS through combining two concepts that utilize porous PLGA MS as a cell delivery system for loading large amounts of chemical drugs and subsequently closing large pores on the MS to control drug release.

## 2. Materials and Methods

### 2.1. Materials

PLGA (38–54 kDa; lactide/glycolide ratio = 50/50), sodium alginate, and calcium chloride were purchased from Sigma-Aldrich (St. Louis, MO, USA). Donepezil hydrochloride was obtained from Acros Organics (Geel, Belgium). Ammonium bicarbonate, dichloromethane, sodium hydroxide solution (1 N), and ethyl alcohol were purchased from Samchun Chemicals (Pyeongtaek, Korea). Polyvinyl alcohol (66 kDa) was obtained from Duksan Pure Chemicals Co., Ltd. (Ansan, Korea).

### 2.2. Preparation of Porous PLGA MS

PLGA MS were prepared by using a double emulsion solvent evaporation method under different conditions (Table 1) [34]. Briefly, PLGA was dissolved in dichloromethane at a concentration of 4% (*w*/*v*). Different concentrations of an aqueous ammonium bicarbonate solution were added to the PLGA solution at an aqueous to organic phase volume ratio of 1:10. The mixture was homogenized at 7200 rpm using a homogenizer to form a water-in-oil emulsion. The water-in-oil emulsion (5.5 mL) was then added into 200 mL of 0.1% (*w*/*v*) polyvinyl alcohol solution and agitated at 800 rpm for 10 h using an overhead stirrer to prepare a water-in-oil-in-water (W/O/W) emulsion. Agitation of the double emulsion was continued for 10 h to evaporate dichloromethane. The PLGA MS with diameters of 45–150 μm were separated using sieves and washed with distilled water. To open the pores formed in the MS, the MS were immersed in 0.05 N sodium hydroxide solution containing 5% (*v*/*v*) ethanol and shaken at 150 rpm for 30 min. After washing the PLGA MS with distilled water, the MS were freeze-dried. For the preparation of drug-loaded PLGA MS, the MS (30 mg) were pre-wetted by immersion into 10 mL of 10% (*v*/*v*) ethanol for 3 min. The MS were then washed with distilled water and immersed in 2 mL of aqueous donepezil solutions of different drug concentrations ranging from 1 mg/mL to 4 mg/mL with shaking at 150 rpm for 6 h. Finally, the MS were separated by centrifugation and freeze-dried.

### 2.3. Evaluation of Encapsulation Efficiency (EE) and Loading Capacity (LC) in Porous PLGA MS

To evaluate the drug EE and LC in the porous MS, the amounts of unloaded drug in the supernatants after centrifugation during the preparation of porous PLGA MS were determined using high-performance liquid chromatography (HPLC), as described below in detail. The amounts of loaded drug in the MS were obtained by subtracting the amounts of unloaded drug from the total amounts of drug taken for encapsulation. EE and LC in the porous MS were calculated using the following equations:

(1) $$\mathrm{EE}\;\left(\%\right)=\frac{\mathrm{Amount}\;\mathrm{of}\;\mathrm{drug}\;\mathrm{loaded}\;\mathrm{in}\;\mathrm{MS}}{\mathrm{Total}\;\mathrm{Amount}\;\mathrm{of}\;\mathrm{drug}}\;\times \;100$$

(2) $$\mathrm{LC}\;\left(\%\right)=\frac{\mathrm{Amount}\;\mathrm{of}\;\mathrm{drug}\;\mathrm{loaded}\;\mathrm{in}\;\mathrm{MS}}{\mathrm{Weight}\;\mathrm{of}\;\mathrm{drug}\;\mathrm{loaded}\;\mathrm{MS}}\;\times \;100$$

### 2.4. HPLC Analysis of Donepezil

The levels of donepezil in samples were measured using HPLC (Waters, Milford, MA, USA), as described previously, but with a minor modification [8]. The HPLC system was composed of an ultraviolet/visible detector (Waters 2489), binary HPLC pump (Waters 1525), and autosampler (Waters 2707). The mobile phase consisted of 0.5% (*v*/*v*) glacial acetic acid and 1% (*v*/*v*) triethylamine in water and methanol (35:65 *v*/*v*). The sample was injected into a Hypersil Gold C-18 column (Thermo Fisher Scientific, 4.6 × 250 mm, 5 µm) at a flow rate of 1 mL/min and monitored at a wavelength of 271 nm.

### 2.5. Preparation of PLGA MS with Closed Pores

The pores of porous PLGA MS were closed by using a spray-ionotropic gelation method. Porous donepezil-loaded PLGA MS were added to 5 mL of sodium alginate solutions of different alginate concentrations (Table 1) and gently mixed. The suspensions were sprayed into 2% (*w*/*v*) calcium chloride solution using a spray machine with a 0.8 mm two-fluid nozzle at a pressure of 1.5 kg/cm^2^. After spraying, the PLGA MS with closed pores were collected by a sieving technique, washed with distilled water, and then freeze-dried.

### 2.6. Observation of Morphology of Porous or Pore-Closed PLGA MS

The morphology of the MS was examined using a scanning electron microscope (SEM) (S-3400N, Hitachi, Tokyo, Japan). The PLGA MS were fixed on a metal stub using double-sided carbon tapes and observed at an accelerating voltage of 2.0 kV.

### 2.7. Assessment of Pore Size and Porosity of Porous PLGA MS

Average pore size and porosity of porous PLGA MS were analyzed using a mercury intrusion porosimeter (Autopore IV 9500, Micromeritics, Norcross, GA, USA). Mercury was filled into the pore spaces of the PLGA MS at pressures ranging from 0.4 psia to 60,000 psia in the intrusion chamber. The equilibration time for obtaining each data point was set at 10 s. The average pore size and porosity of the MS were calculated. Additionally, the apparent density of the MS was measured.

### 2.8. Evaluation of Particle Size Distribution of Porous or Pore-Closed PLGA MS

The particle size distributions of porous or pore-closed PLGA MS were analyzed by using a Mastersizer 3000 (Malvern instruments Ltd., Malvern, United Kingdom). An appropriate amount of porous PLGA MS was dispersed in ethanol. The assessment of the particle size distribution was conducted when laser obscurations reached between 2% and 3%. Volume-weighted size distributions were obtained, and the particle diameters representing the 10th, 50th, and 90th percentiles of the volume-weighted size distributions were defined as d_10_, d_50_, and d_90_, respectively. Span index, a factor indicating the width of distribution, was calculated as follows: (d_90_ − d_10_)/d_50_.

### 2.9. Viscosity Measurements of Spraying Suspensions Containing PLGA MS

The viscosity of spraying suspensions containing PLGA MS was evaluated using a rotational rheometer (HAAKE RheoStress 1, Thermo Fisher Scientific Inc., Waltham, MA, USA) equipped with a 35 mm parallel plate. The gap between the plate and stage was set at 1 mm. Each test sample was loaded on the rheometer plate at 20 °C and allowed to equilibrate for 5 min prior to the viscosity evaluation. The viscosity measurements were performed at a constant stress of 30 Pa.

### 2.10. Injectability

Injectability of suspensions containing PLGA MS with closed pores was assessed by a previously described method [39], with a slight modification by using a texture analyzer (TA plus, Lloyd Instruments, Bognor Regis, United Kingdom). Each final formulation (30 mg) was suspended in distilled water (2 mL) and loaded into a 5 mL syringe with a 32 mm 22-gauge needle (inner diameter of 410 µm). The syringe was tightly fixed at a clamp stand with its needle pointing downwards. The plunger end of the syringe was placed in contact with a stainless-steel probe connected to a 10 N loading cell. The loading force to expel the contents from the syringe was measured at a crosshead speed of 1 mm/s. The injectability parameters, such as plunger-stopper break-loose force (PBF), maximum force (*F*_max_), and dynamic glide force (DGF), were determined from the force-time plot [40].

### 2.11. In Vitro Drug Release of Porous or Pore-Closed PLGA MS

Donepezil-loaded porous or pore-closed PLGA MS (30 mg) were added to phosphate-buffered saline (20 mL) of pH 7.4 and shaken at 70 rpm at a constant temperature at 37 °C. After predetermined time intervals, aliquots of 500 μL were withdrawn and filtered through a 0.45 μm polyvinylidene fluoride syringe filter. Fresh medium was added to the dissolution medium to maintain the initial volume. Levels of donepezil in the aliquots of the dissolution medium were determined using HPLC, as described in Section 2.4.

### 2.12. Prediction of Plasma Drug Concentration Profiles by Using Convolution Technique

In order to avoid animal experiments at this initial stage of development, plasma drug concentration profiles after intramuscular administration of the PLGA MS were predicted by using the convolution technique, a method used for predicting in vivo pharmacokinetic behavior and in vitro–in vivo correlation, as described previously [41,42]. We assumed 1.58 mg PLGA MS (corresponding to 0.25 mg donepezil) for intramuscular administration into the human body. The dose was determined by referring to a previously demonstrated donepezil depot injection formulation from a pharmaceutical company that used donepezil base 0.27 mg as an active pharmaceutical ingredient [43]. In this study, the convolution approach was used on the basis of the in vitro drug release profiles and pharmacokinetic parameters established from previous studies of commercially available oral formulation (Aricept) [10,44,45]. The reported pharmacokinetic parameters of donepezil for a person of 70 kg body weight were as follows: bioavailability of intramuscular injection was postulated as 100% (*F* = 1); volume of distribution (*V*_d_) was 11.82 L/kg; clearance (*Cl*) was 0.13 L/kg/h; and half-life of elimination (*t*_1/2_) and rate of first order elimination (*k*_e_) were 63 h and 0.011 h^−1^, respectively.

With reference to these pharmacokinetic parameters, the in vitro drug release data we obtained were then converted into plasma drug concentration profiles by using the following steps. First, amounts of drug released within the sampling interval were obtained from the in vitro cumulative drug release profiles. Second, the amounts of drug released within the sampling interval were assumed to have been absorbed into the blood from the intramuscularly injection site and then undergoing first order elimination kinetics. Third, the total amount of drug existing in the blood was obtained by summing all the calculated amounts of drug released within sampling interval for every time. Finally, the expected plasma drug concentrations were calculated by dividing the total amount of drug existing in the blood by the volume of distribution and body weight.

### 2.13. Statistical Analysis

All experiments were conducted in triplicate and expressed as a mean ± standard deviation (SD), where appropriate. All data were collated and analyzed with Excel software. Means were analyzed using one-way analysis of variance and Student’s *t*-test. A *p*-value < 0.05 was considered significant.

## 3. Results and Discussion

### 3.1. Characterization of Porous PLGA MS

#### 3.1.1. Surface Morphology and Pore Characteristics of Porous PLGA MS

**Figure 1:**
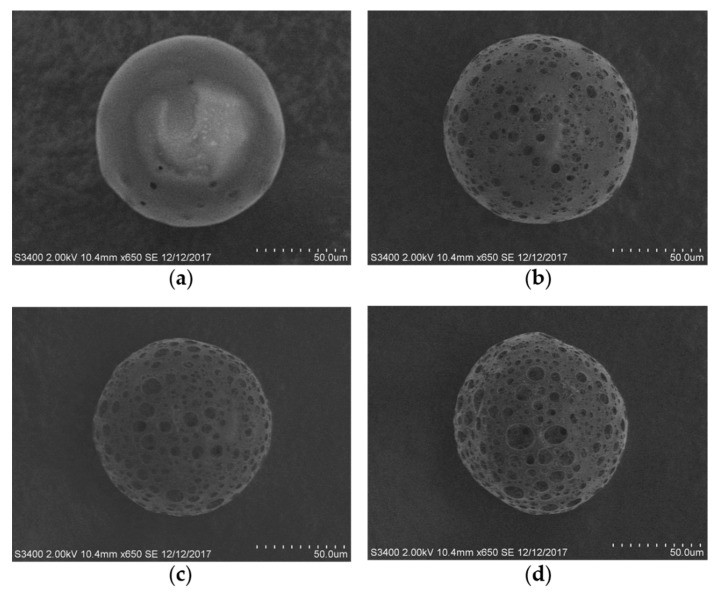
Scanning electron microscopy images of porous poly(lactide-*co*-glycolide) (PLGA) microspheres (MS) from (**a**) F1, (**b**) F2, (**c**) F3, and (**d**) F4. MS were fabricated as stable spheres. Larger pores were formed as the concentration of ammonium bicarbonate increased, due to its ability to generate gas inside MS.

Upon dissolving in water, ammonium bicarbonate produces ammonia and carbon dioxide gases, which expand the inner aqueous phase droplets in the double emulsion, resulting in increased porosity and pore size even after the hardening of the MS [31,46,47]. To fabricate MS with different pore sizes and porosities, we used aqueous solutions of various concentrations of ammonium bicarbonate as the inner aqueous phase of the W/O/W emulsion. The porous structure of the PLGA MS was observed using SEM. PLGA MS prepared using the inner aqueous phase devoid of ammonium bicarbonate exhibited few pores on their surface, whereas the MS fabricated using ammonium bicarbonate solutions showed numerous and large pores (Figure 1). The number of pores on the surface of the MS and the pore sizes were greater when the concentration of ammonium bicarbonate in the inner aqueous phase was higher, which was consistent with the findings of previous studies [34,48,49].

The uniformity of pore structure decreased with increasing the concentration of ammonium bicarbonate in the inner aqueous phase (Figure 1). The reason for this was demonstrated by the previous study [50]. During the solvent evaporation, the ammonium bicarbonate not only expanded the dimension of the inner aqueous phase droplets but also generated the small gas-filled bubble surrounding the inner aqueous phase droplets. This small gas bubble prevented the inner aqueous phase droplets from coalescing each other, thereby leading to a formation of adequate porous structures in the PLGA MS. In the porous structure, the large pores were produced from the inner aqueous phase droplets, and the small pores located between the large pores were originated from evolved gas bubbles. The size difference between the inner water phase droplets and the small gas bubbles could be smaller at the low concentration condition of ammonium bicarbonate, but it would become larger at the high concentration condition of ammonium bicarbonate. Therefore, pore size distribution consisting of large and small pores became broad when the concentration of ammonium bicarbonate was increased in the inner aqueous phase.

The pore size and porosity of porous PLGA MS were examined by a mercury intrusion porosimeter. Table 2 shows average pore size, porosity, and apparent density of the MS. The pore sizes and porosities of the PLGA MS increased considerably with increasing concentrations of ammonium bicarbonate in the inner aqueous phase. Thus, pore size and porosity of the PLGA MS could be adjusted by changing the concentration of ammonium bicarbonate in the inner aqueous phase of the W/O/W emulsion. This was important for our objective because PLGA MS presenting large pores and high porosity were expected to accommodate more drugs in the MS, leading to high EE and LC of donepezil in the MS.

#### 3.1.2. EE and LC of Donepezil in Porous PLGA MS

**Figure 2:**
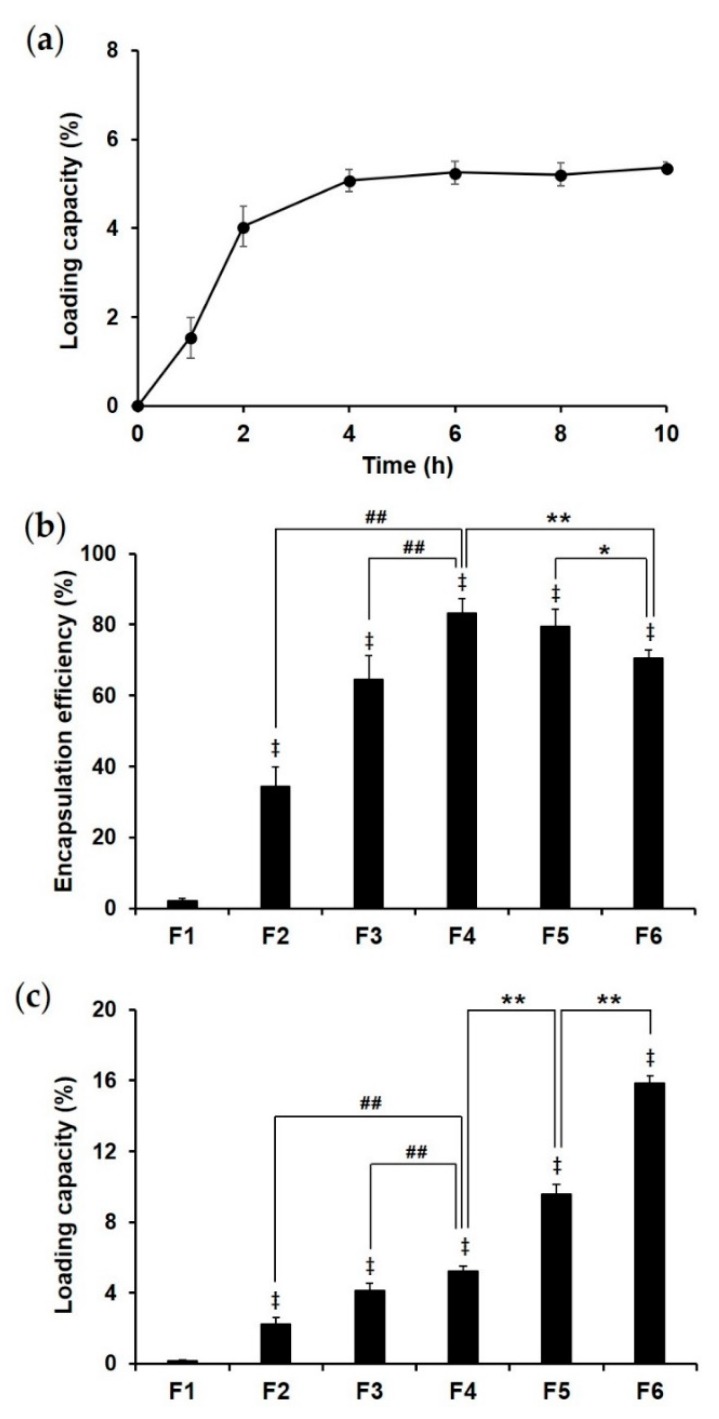
(**a**) Loading capacity (%) in porous PLGA MS (F4) as a function of time (hours). Drug loading capacity (%) reached equilibrium at 6 h. (**b**) Encapsulation efficiency and (**c**) loading capacity of formulations F1–F6. The values are expressed as mean ± SD (*n* = 3). ^‡^ *p* < 0.01 compared to F1, ^##^ *p* < 0.01 compared to F4, * *p* < 0.05 compared to F6, ** *p* < 0.01 compared to F6. PLGA, poly(lactide-*co*-glycolide); MS, microspheres.

To formulate donepezil-loaded, porous PLGA MS with maximum EE and LC, we fabricated the MS by varying experimental conditions such as drug loading time, porosity of the MS, and the drug concentration in aqueous solutions used for drug loading. LC in the porous MS (F4) gradually increased over a period of 6 h, after which there was no significant change in EE or LC until 10 h (Figure 2a). This could be attributed to the initial migration of the drug solution via pores into the MS until the MS were wet enough, after which the donepezil could be loaded quickly until saturation. Therefore, we chose 6 h as the optimal drug loading time to maximize EE and LC in the MS. We also found high EE (83.2% ± 4.32%) and LC (5.25% ± 0.26%) of donepezil in F2–F4 MS as the porosity of these MS was higher (Figure 2b,c). These results clearly show that the PLGA MS with big pores and high porosity possess a high capacity in containing drug solution.

We subsequently examined the effect of donepezil concentration in the solutions on EE and LC in F4–F6 MS. With increasing drug concentration in the solution, we noted a slight decrease in EE and a considerable increase in LC in the porous MS. The slight decrease in EE could be attributed to the higher increments in the amount of unloaded donepezil compared to the amount of loaded donepezil. In contrast, the remarkable increase in LC could have been because of the extremely low density of the porous MS. Although the EE of F6 was slightly lower than that of F4, F6 had the LC value of 15.84% ± 0.44%, which is optimal for intramuscular injectable depot systems because high LC implies that the formulation can be injected with small amounts of MS for the same dose. Therefore, F6 was chosen as the optimal formulation that was to be used for the next step, namely, pore-closing via a spray-ionotropic gelation method.

### 3.2. Characterization of PLGA MS with Closed Pores

#### 3.2.1. Surface Morphology of PLGA MS with Pores Closed by Calcium Alginate Coating

**Figure 3:**
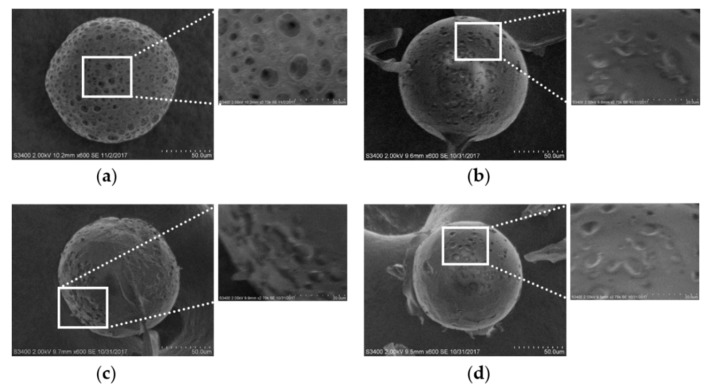
Surface morphology of (**a**) PLGA MS without calcium alginate coating (F6) and the MS with pores closed using sodium alginate solutions of different concentrations of (**b**) 1% (F7), (**c**) 1.5% (F8), and (**d**) 2% (F9) observed by scanning electron microscopy. PLGA, poly(lactide-*co*-glycolide); MS, microspheres.

After we selected F6 as the optimal porous PLGA MS formulation, we applied spray-ionotropic gelation to close the pores of porous PLGA MS in F6. All the porous MS examined using SEM (Figure 3) showed intact spherical shapes, indicating that the spraying conditions used in the pore-closing procedure did not cause considerable physical damage to the MS. Figure 3b–d shows calcium alginate coating closing the pores on the surface of the MS, whereas MS with open pores (without the calcium alginate coating) on their surface are shown in Figure 3a. The number of the pores closed with the alginate coating increased with increasing concentration of alginate used in the pore-closing process. Furthermore, the surface of the MS became smoother with increasing alginate concentration, implying that the thickness of the alginate coating formed on the surface of the MS had increased with increasing alginate concentration.

#### 3.2.2. Particle Size of PLGA MS with Closed Pores and Viscosity of Spraying Suspensions

**Figure 4:**
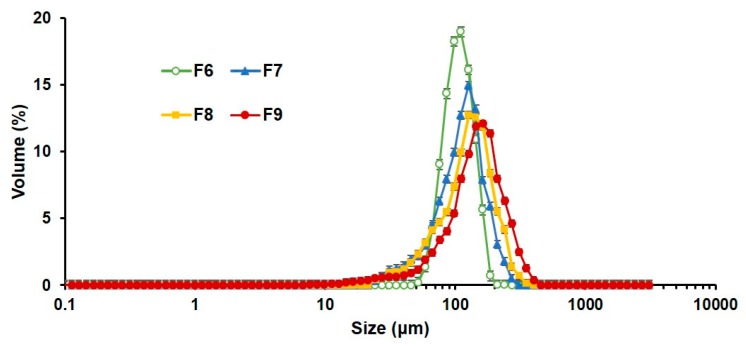
Particle size distribution of PLGA MS F6 to F9 (mean ± SD, *n* = 3). PLGA, poly(lactide-*co*-glycolide); MS, microspheres.

Figure 4 presents the particle size distribution of PLGA MS with pores closed by alginate coating (F6–9). Curves of F7, F8, and F9 were overlaid with the graph of uncoated MS, F6. The average particle size (d_50_) increased with increasing sodium alginate/PLGA ratios, as high sodium alginate/PLGA ratios implied that higher amounts of sodium alginate underwent ionotropic gelation with calcium chloride, resulting in thicker calcium alginate coating and a higher average particle size (Table 3). Solutions or suspensions of higher viscosity form larger droplets when sprayed [51]. Hence, formulations containing high levels of sodium alginate might have had higher viscosity, producing larger droplets when they were sprayed through a nozzle compared to those containing low levels of sodium alginate. The pores of these large droplets probably closed immediately upon contact with calcium chloride solution, owing to ionotropic gelation of alginate by calcium. Therefore, the increased droplet size caused by the higher viscosity of the formulations probably resulted in the overall growth of the particle size. Besides the particle size, all the formulations showed relatively narrow distribution with span values ranging from 0.67 to 1.23. Increasing the amount of sodium alginate caused higher viscosity of the spraying suspension, leading to a slight broadening of the particle size distribution, which was consistent with the findings of a previous report [52].

#### 3.2.3. Injectability of PLGA MS with Closed Pores

**Figure 5:**
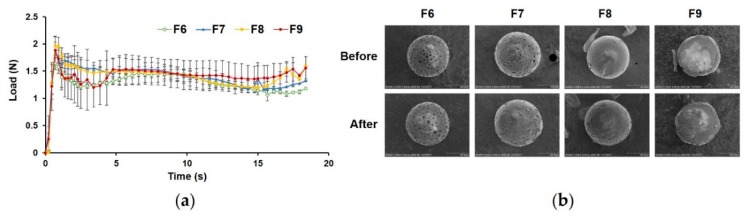
Injectability of porous (F6) and pore-closed (F7–9) microspheres (MS) suspended in distilled water. (**a**) Loading force on plunger required to expel the suspension of the formulations from syringes with a 32 mm 22-gauge needle as a function of time at a crosshead speed of 1 mm/s (mean ± SD, *n* = 3). All formulations showed a peak representing the plunger-stopper break-loose force (PBF), followed by a relatively stable dynamic glide force (DGF) phase. No drastic changes in load were observed after the peak, indicating that the MS did not plug the needle or aggregate in the syringe. (**b**) Morphology of the MS observed before and after the injection through syringes with a 32 mm 22-gauge needle. No remarkable changes on the surface of the MS were observed.

Figure 5a depicts the loading force-time profiles during injection of the pore-closed MS suspensions through syringes with 22-gauge needles, showing an initial PBF, followed by the DGF phase. PBF, *F*_max_, and DGF of the MS suspensions (F6–9) were all significantly higher than those of the blank distilled water sample. For the pore-closed MS suspensions (F7–9), the PBF, *F*_max_, and DGF values were not statistically different from each other (Table 4). Although greater force was needed to inject the pore-closed PLGA MS suspensions compared to the blank sample, all formulations were reasonably acceptable for manual use by clinicians according to the standard of the International Organization for Standardization (ISO) [53]. In addition, when comparing the morphology of the MS before and after injection using SEM (Figure 5b), there were no noticeable changes in the surface of the MS, indicating that needle gauge, volume, and size of the syringe were suitable for clinical use of these formulations.

#### 3.2.4. In Vitro Release

**Figure 6:**
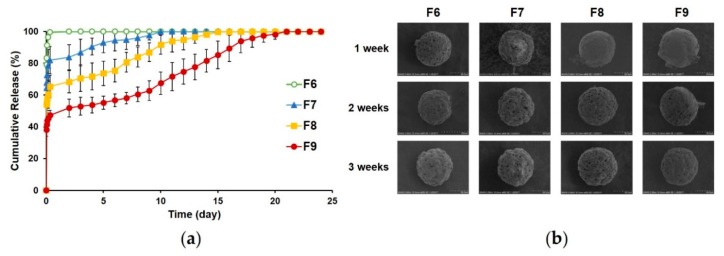
In vitro release of porous (F6) and pore-closed (F7–9) PLGA MS. (**a**) Cumulative release data (mean ± SD, *n* = 3) and (**b**) morphology of each formulation during in vitro release test. PLGA, poly(lactide-*co*-glycolide); MS, microspheres.

Cumulative drug release data from porous or pore-closed MS are shown in Figure 6a. We examined in vitro release using PBS (pH 7.4) as a release medium to simulate the conditions of the human muscle, in which the interstitial pH is 7.4 [54]. The uncoated, porous PLGA MS (F6) released most of the drug within 8 h (99.5% ± 0.81%). The pore-closed PLGA MS formulations (F7–9) showed an initial burst release in 8 h (81.9% ± 8.53% for F7, 65.4% ± 6.94% for F8, and 47.3% ± 2.95% for F9), which was much slower than that of F6. This difference could be attributed to the calcium alginate coating layer acting as a barrier to the diffusion of the drug, retarding the rapid release of the drug encapsulated in the MS. The initial burst release of the formulations was followed by a sustained release of the drug over 20 days. In fact, the formulation containing higher alginate content showed thicker calcium alginate layers, and it presented a much slower, sustained release pattern. These results indicate that the release pattern of donepezil from the pore-closed PLGA MS could be controlled by changing the thickness of the calcium alginate layers, as was previously reported for other formulations [55,56].

Morphological changes were also observed in each formulation during the in vitro release experiment (Figure 6b). Open pores were observed in F6 in the beginning of the in vitro release experiment, and gradual erosion was observed in the PLGA MS structure over the course of the in vitro release experiment. The erosion became more obvious after 2 weeks, although the pore structure of the MS was still maintained. After 1 week, F7 showed an open porous structure, whereas F8 and F9 still had closed pores. This indicated that the calcium alginate coating on F7 MS was degraded in 1 week, but that of F8 and F9 was partially maintained. These observations were consistent with the release profiles, wherein the release of F7 was almost complete in 1 week, whereas F8 and F9 showed sustained release beyond this period. Almost all the pores of F8 were observed as being open after 2 weeks, whereas the pores of F9 were partially closed. After 3 weeks, all formulations showed open pores on their surface. From these SEM images and the in vitro release profiles, it can be concluded that the rate of release of the drug increased as the number of open pores increased, which indicates that the degradation of the calcium alginate layers controlled the release patterns of the PLGA MS formulations. Thus, adjustment of the thickness of the calcium alginate coating during the fabrication of the PLGA MS formulations can be used as a strategy to control the release of the drug.

Furthermore, we assumed the elimination time of the PLGA MS formulations in the human body on the basis of our SEM observation data and previous reports [22,34,57]. It is well known that PLGA of an equal lactide (L) to glycolide (G) ratio degrades into monomers, lactic acids, and glycolic acids within 12 weeks. After the degradation, the monomers are further metabolized and subsequently eliminated from the body. Our previous work also showed that most of the PLGA MS, designed for a cell delivery system, composed of PLGA of a low molecular weight (30 kDa) and an equal L/G ratio degraded within 12 weeks [34]. It was also reported that the formulation composed of PLGA of a high molecular weight (135 kDa) and an equal L/G ratio degraded more than 90% of the PLGA scaffold in 12 weeks [57]. In our study, most of the calcium alginate layers on the PLGA MS dissolved in the PBS within 4 weeks, and simultaneously the PLGA (38–54 kDa, L/G ratio = 50/50) components in the formulation were expected to be degraded in 12 weeks and subsequently eliminated from the human body. Therefore, the elimination time of the pore-closed PLGA MS in the human body was considered as 12–16 weeks, and further studies still need to be conducted on controlling the degradation of the PLGA MS.

Driving forces for the PLGA MS with closed pores to release the encapsulated drug appeared to be the following three mechanisms: the degradation of the PLGA, the dissolution of the calcium alginate membrane, and the diffusion of the drug at the calcium alginate membrane. However, as the SEM observation showed that the pore structure of the PLGA MS remained until the whole degradation of the calcium alginate coatings, the drug release was considered to be mainly controlled by the calcium alginate layers. As for the initial burst release, we could infer that the drug loaded in the pore structure of the PLGA MS with high concentrations might be initially released from the partly thinly coated or partly non-coated pore space of the calcium alginate membrane.

#### 3.2.5. Prediction of Plasma Drug Concentrations for PLGA MS Depot Formulations

**Figure 7:**
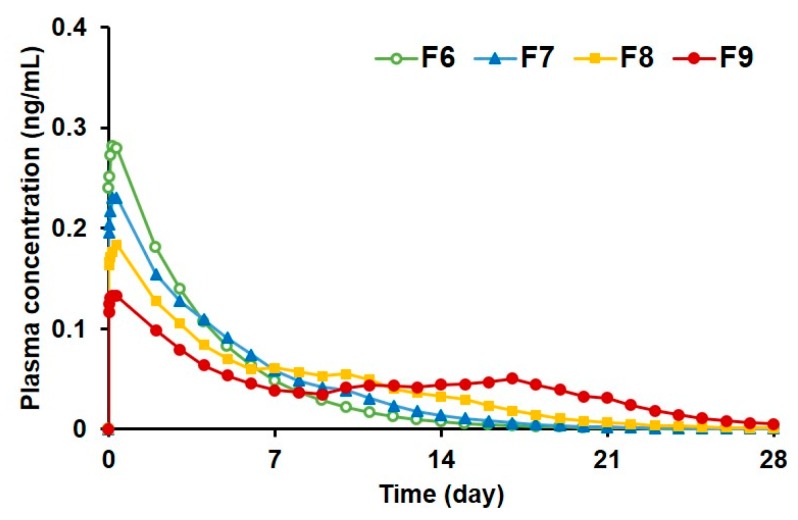
Predicted plasma concentration profiles of donepezil after intramuscular injection of the PLGA MS (F6–9) using the convolution technique. The pore-closed PLGA MS (F7–9) exhibited superior sustained release profiles compared to the porous PLGA MS (F6).

Figure 7 shows the predicted plasma concentration–time profiles obtained by using the convolution method [41,42], on the basis of the in vitro drug release data and the pharmacokinetic parameters established from previous clinical studies [10,44,45]. The peak concentration (*C*_max_) was predicted to be reached within the first day of injection for all the porous (F6) and pore-closed PLGA formulations (F7–9) due to the initial burst release of donepezil, after which the predicted drug concentrations declined gradually. As the alginate concentration used in the PLGA MS increased, the predicted *C*_max_ decreased, whereas the predicted mean residence time (MRT) values increased (Table 5). These data imply that the alginate coatings on the PLGA MS successfully retarded the initial burst release of the drug from the big pores and prolonged the sustained release profiles of the PLGA MS. In fact, the predicted MRT value of the pore-closed PLGA MS (F9) was extended to 10.14 days, which was 2.7 times longer than that of the porous PLGA MS (F6). Therefore, compared to the once-a-day dosing regimen of commercially available oral dosage forms, the pore-closed PLGA MS could be expected to greatly reduce the frequency of administration, leading to enhanced patient compliance.

## 4. Conclusions

In this study, PLGA MS with large pores containing donepezil were prepared with high EE and LC. After the optimization of various conditions for preparation of the porous PLGA MS, LC was considerably enhanced up to 15.84% ± 0.44% in the porous PLGA MS. This implies that a smaller amount of these MS could be used for the same dose in depot injections. However, an initial burst release of donepezil from the large pore spaces was observed. To overcome this problem, we used sodium alginate as a pore-closing agent, which forms a cross-linked gel with calcium chloride. The resulting calcium alginate coating on the PLGA MS with big pores was visualized by SEM and characterized using particle size measurements. The PLGA MS with closed pores demonstrated in vitro sustained release of donepezil that lasted over 3 weeks. Further, the plasma drug concentration profiles were predicted by using the convolution technique, assuming intramuscular injection in the human body. In the prediction, the pore-closed PLGA MS exhibited greatly reduced initial burst release and enhanced sustained-release profiles for 2–4 weeks, indicating that the calcium alginate coating on the PLGA MS with big pores successfully retarded the initial burst release of the PLGA MS and maintained the effective concentration for a prolonged time. Therefore, our results suggest that the PLGA MS with alginate-coated large pores hold promise for the management of dementia with high patient compliance.

## Figures and Tables

**Table 1:** Preparation conditions of porous poly(lactic-*co*-glycolic acid) (PLGA) microspheres (MS) and pore-closed PLGA MS.

PLGA MS	Formulation Code	PLGA (%, *w*/*v*)	Ammonium Bicarbonate (%, *w*/*v*)	Donepezil Hydrochloride (mg/mL)	Sodium Alginate (%, *w*/*v*)
Porous PLGA MS	F1	4	-	1	-
	F2	4	1	1	-
	F3	4	2	1	-
	F4	4	4	1	-
	F5	4	4	2	-
	F6	4	4	4	-
Pore-closed PLGA MS	F7	4	4	4	1
	F8	4	4	4	1.5
	F9	4	4	4	2

PLGA, poly(lactide-*co*-glycolide); MS, microspheres.

**Table 2:** Pore characteristics of formulations F1–F4 of porous PLGA microspheres.

Parameter	F1	F2	F3	F4
Average pore size (µm)	1.45 ± 0.38	2.13 ± 0.64	2.70 ± 0.37	2.86 ± 0.33
Porosity (%)	8.93 ± 0.91	29.9 ± 7.26	47.3 ± 8.11	63.4 ± 9.08
Apparent density (g/mL)	1.74 ± 0.66	1.13 ± 0.20	0.79 ± 0.26	0.43 ± 0.13

The data are presented as mean ± SD, *n* = 3.

**Table 3:** Zero shear viscosity of spraying suspensions and particle size parameters of the PLGA MS (F6–9).

Parameter	F6	F7	F8	F9
ŋ_0_ (mPa·s)	-	48.2 ± 2.57	162.6 ± 3.52	393.8 ± 5.49
d_10_ (μm)	71.2 ± 0.40	56.1 ± 1.28	57.0 ± 1.81	64.4 ± 3.30
d_50_ (μm)	99.8 ± 1.73	108.6 ± 5.96	120.6 ± 4.92	138.9 ± 3.42
d_90_ (μm)	138.2 ± 3.95	160.2 ± 1.44	194.2 ± 6.92	235.5 ± 9.08
Span	0.67 ± 0.02	0.96 ± 0.07	1.14 ± 0.03	1.23 ± 0.09

The data are expressed as mean ± SD, *n* = 3. Zero shear viscosity (ŋ_0_); particle diameters at 10 (d_10_), 50 (d_50_), and 90 (d_90_) percentage cumulative volume; span, (d_90_ − d_10_)/d_50_.

**Table 4:** Injectability parameters of the formulations suspended in distilled water with 22-guage needles (mean ± SD, *n* = 3).

Formulation	PBF (N)	*F*_max_ (N)	DGF (N)
F6	1.65 ± 0.09	1.53 ± 0.09	1.29 ± 0.03
F7	1.90 ± 0.24	1.76 ± 0.12	1.47 ± 0.09
F8	1.97 ± 0.17	1.74 ± 0.10	1.41 ± 0.13
F9	1.89 ± 0.17	1.86 ± 0.07	1.44 ± 0.14

PBF, plunger-stopper break loose force; *F*_max_, maximum force; DGF, dynamic glide force.

**Table 5:** Predicted pharmacokinetic parameters for the PLGA MS formulations F6–9.

Parameter	F6	F7	F8	F9
*C*_max_ (ng/mL)	0.28	0.23	0.18	0.13
AUC_0-∞_ (ng·day/mL)	1.16	1.19	1.21	1.24
MRT (day)	3.75	4.75	6.64	10.14

*C*_max_, peak plasma drug concentration; AUC, area under the curve; MRT, mean residence time.
